# Case Report: Multimodal immunotherapeutic regimen (envafolimab + chemotherapy + radiotherapy) for four synchronous primary malignant neoplasms: a case report of 33-month survival and implications for tumor immunology

**DOI:** 10.3389/fonc.2025.1727323

**Published:** 2026-01-08

**Authors:** Yadong Liu, Yayu Zhang, Yu Zhang, Xuejuan Duan, Zhanjie Gao, Xianbo Zhang, Jing Zhao

**Affiliations:** 1 Hebei General Hospital, Shijiazhuang, China; 2 Fourth Hospital of Hebei Medical University, Shijiazhuang, China

**Keywords:** immunotherapy, multiple primary malignant neoplasms, treatment outcomes, clinical efficacy, chemotherapy

## Abstract

Multiple Primary Malignant Neoplasms (MPMNs) are defined as the presence of two or more independent malignant tumors in the same patient, either simultaneously or sequentially, presenting substantial diagnostic and therapeutic challenges. In recent years, immunotherapy—an emerging therapeutic modality—has shown notable clinical efficacy across a range of malignancies. This article reports a case of a patient with four concurrent primary malignancies who attained long-term survival following treatment with envafolimab injection. Using this case as a foundation, we discuss recent advances in immunotherapy for MPMNs, covering its mechanisms of action, clinical efficacy, therapeutic strategies, and current challenges. The goal is to provide a theoretical basis for clinical practice and identify future research directions.

## Introduction

Multiple Primary Malignant Neoplasms (MPMNs) are defined as the synchronous or metachronous occurrence of two or more histologically distinct and mutually independent malignant tumors in a single patient. With advancements in cancer diagnosis and therapeutic technologies, patient survival has improved, contributing to the rising incidence of Multiple Primary Malignant Neoplasms (MPMNs) (1, 2). The pathogenesis of MPMNs remains incompletely understood but may involve genetic predisposition, environmental factors, lifestyle choices, and treatment-related factors (3). However, there is currently no standard treatment regimen for multiple primary malignant neoplasms (MPMNs). Their complex biological characteristics and heterogeneity limit the efficacy of conventional treatments such as surgery, chemotherapy, and radiotherapy.

Immunotherapy, an emerging cancer treatment approach—works by activating the patient’s immune system to target and eliminate tumors, and it has shown considerable efficacy in the management of various malignancies. However, its use in MPMNs is still in the exploratory stage, and its efficacy and safety warrant further investigation. Through this case report, we seek to explore the role of immunotherapy in MPMNs, analyze its therapeutic mechanisms and clinical outcomes, and offer practical insights to inform clinical practice.

## Case presentation

A 71-year-old female presented to our outpatient clinic on May 3, 2022, with a one-month history of abdominal pain. On physical examination, her body temperature was normal; she reported no cough or expectoration, and bilateral breath sounds were clear. Her abdomen was soft with tenderness in the right lower quadrant, and there was no rebound tenderness, muscle rigidity, or lower extremity edema. The patient had undergone right hip replacement surgery in 2016 following a traffic accident and had no history of smoking, alcohol consumption, or familial genetic disorders.

**Figure 1:**
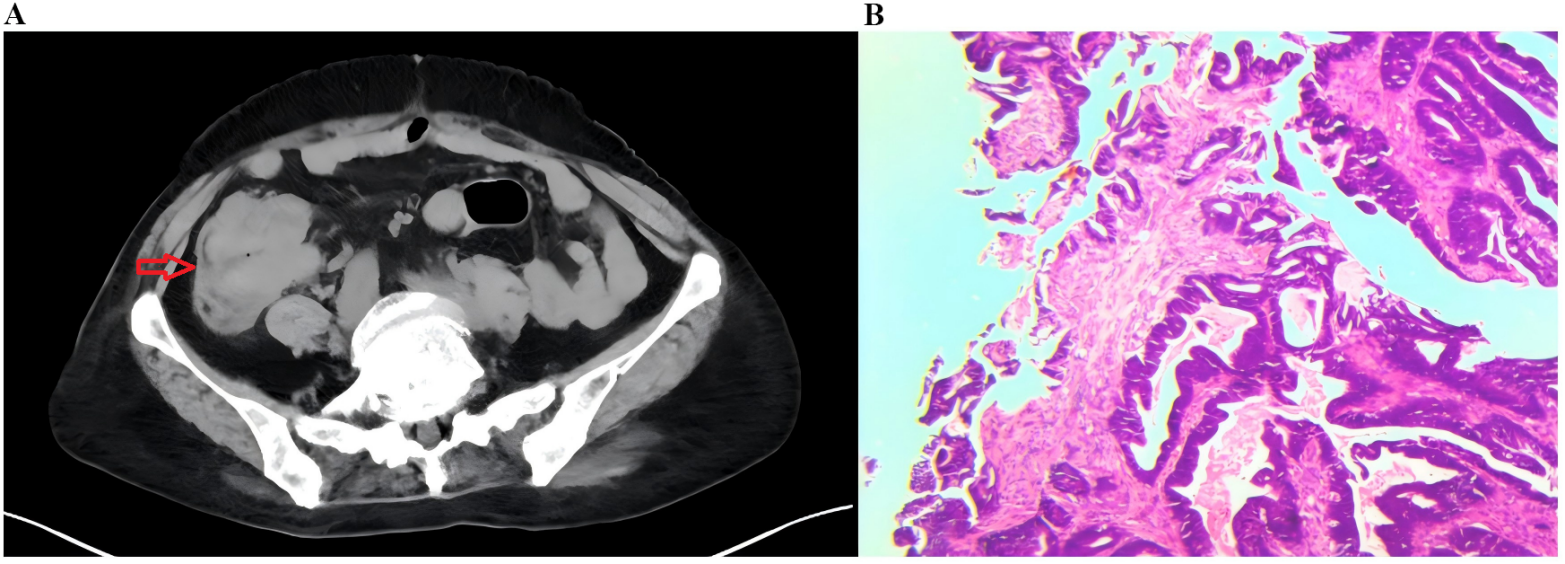
Imaging and pathological findings. **(A)** Axial CT showing a mass in the colon; **(B)** Microscopy revealing adenocarcinoma (H&E stain, ×200).

**Figure 2:**
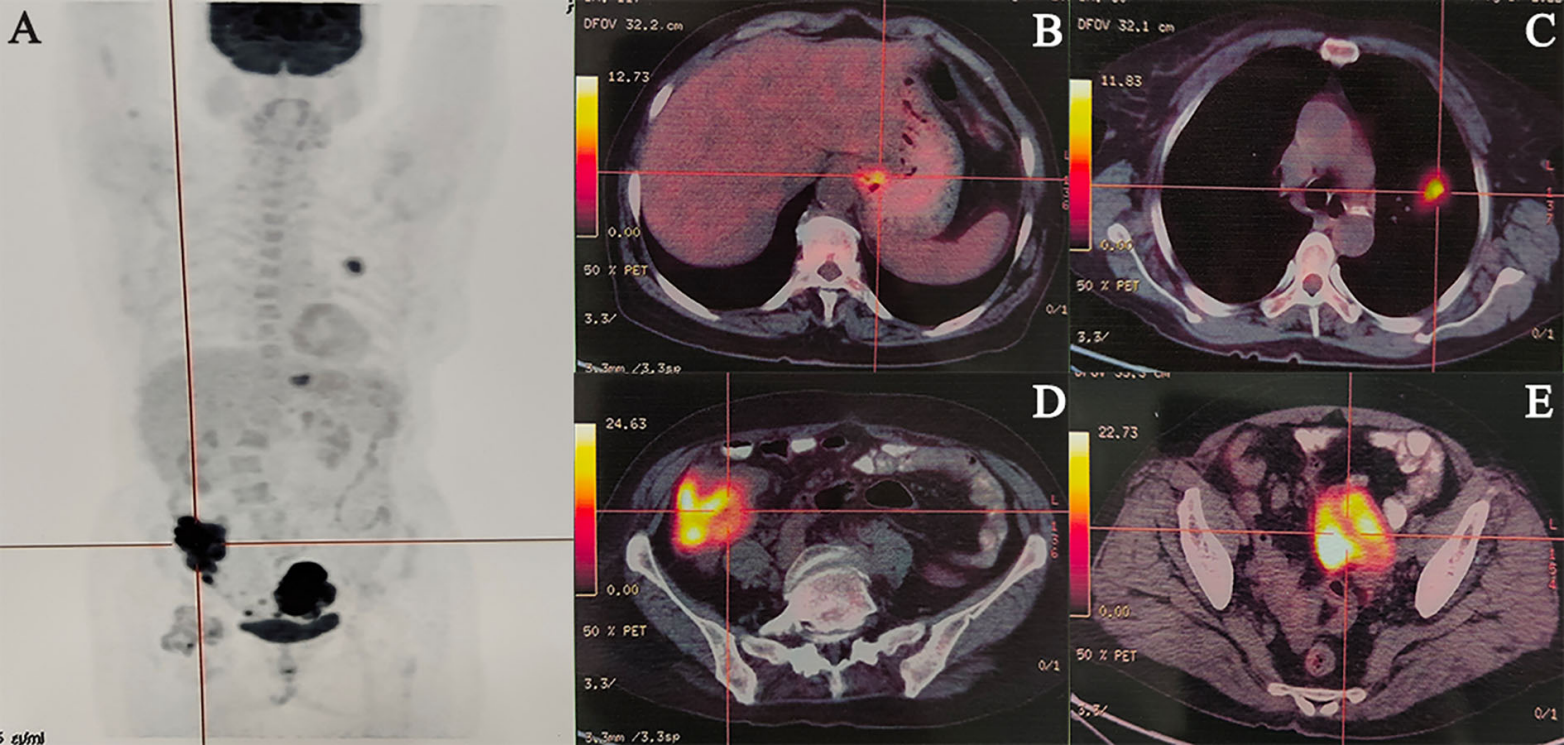
Representative cases of malignancies. **(A)** Maximum Intensity Projection, **(B)** Gastric cardia cancer, **(C)** Left lung cancer, **(D)** Colon cancer, **(E)** Ovarian cancer.

An abdominal computed tomography (CT) scan performed on May 3, 2022, identified a mass in the ascending colon (**Figure 1A**). A subsequent colonoscopy (May 5, 2022) and pathological biopsy confirmed moderately differentiated adenocarcinoma (**Figure 1B**). The patient was admitted for surgical evaluation. Preoperative workup revealed additional masses in the left ovary and left lung, as well as gastric wall thickening at the cardia. Imaging features were inconsistent with metastatic disease. Positron emission tomography-computed tomography (PET-CT; **Figure 2**) demonstrated the following: 1.Hypermetabolic thickening in the ileocecal region, consistent with colorectal cancer; 2. A hypermetabolic soft tissue mass in the left pelvis, consistent with a primary pelvic tumor; 3. A hypermetabolic nodule in the apical-posterior segment of the left upper lung, consistent with invasive lung adenocarcinoma; 4. Hypermetabolic thickening of the gastric wall at the cardia, suspicious for gastric adenocarcinoma.

**Figure 3:**
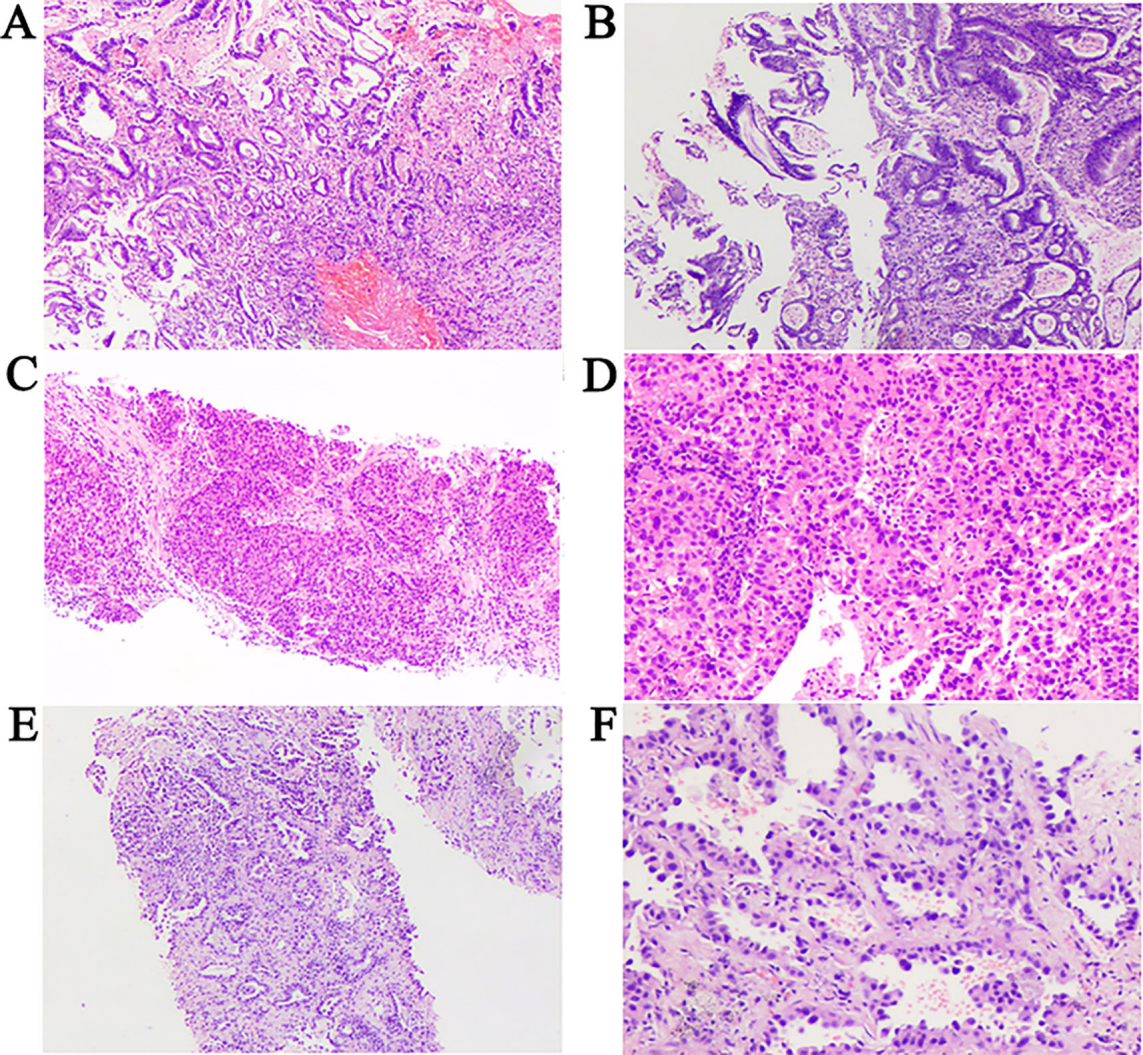
Pathological findings. **(A, B)** Pathological results cardiac cancer. H&E staining of biopsy samples (100×) magnification. **(C)** Pathological results of ovarian cancer. H&E staining of biopsy samples (40×) magnification. **(D)** Pathological results of ovarian cancer. H&E staining of biopsy samples (100×) magnification. **(E)** Pathological results of lung cancer. H&E staining of biopsy samples (40×) magnification. **(F)** Pathological results of lung cancer. H&E staining of biopsy samples (200×) magnification.

Pathological assessment confirmed the following: 1.Gastroscopic biopsy of the gastric cardia mass revealed moderately to poorly differentiated adenocarcinoma (**Figures 3A, B**); 2. Ultrasound-guided biopsy of the left ovarian mass confirmed ovarian carcinoma (**Figures 3C, D**); 3. CT-guided biopsy of the left lung mass confirmed lung adenocarcinoma (**Figures 3E, F**). Staging of all tumors and the corresponding staging criteria are provided in the **Supplementary Materials** (**Supplementary Table S1**).

Comprehensive tumor-derived DNA next-generation sequencing (NGS) was performed on pretreatment biopsy specimens of the primary colorectal adenocarcinoma. The panel evaluated microsatellite instability (MSI) status, tumor mutational burden (TMB), and somatic alterations in the *KRAS*, *NRAS*, *BRAF*, and *PIK3CA* genes. Results showed high MSI (MSI-H), a TMB of 8 mutations/Mb, and no actionable driver mutations.

Given the presence of four synchronous primary tumors, germline analysis was performed using a multi-gene hereditary cancer panel (including *MLH1*, *MSH2*, *MSH6*, *PMS2*, *APC*, *BRCA1*, *BRCA2*, and *TP53*) on peripheral blood lymphocytes. No pathogenic germline variants were identified, thereby ruling out Lynch syndrome and confirming a sporadic etiology for the MPMNs. Targeted gene testing of the lung adenocarcinoma (for *EGFR*, *ALK*, *ROS1*, *RET*, and *MET*) revealed no clinically actionable mutations.

Immunohistochemical (IHC) staining of the lung tumor showed TTF-1 positivity and NapsinA positivity, confirming a primary lung adenocarcinoma; CK20 negativity and CDX-2 negativity ruled out a metastatic colonic origin. All lesions were thus confirmed to be independent primary malignancies. A multidisciplinary team (MDT) consultation concluded that radical resection of all four tumors would pose excessive morbidity risks. Instead, the patient received six cycles of combined immunotherapy and chemotherapy, followed by one year of immunotherapy maintenance, with regular surveillance and adjunctive interventions administered as needed.

**Figure 4:**
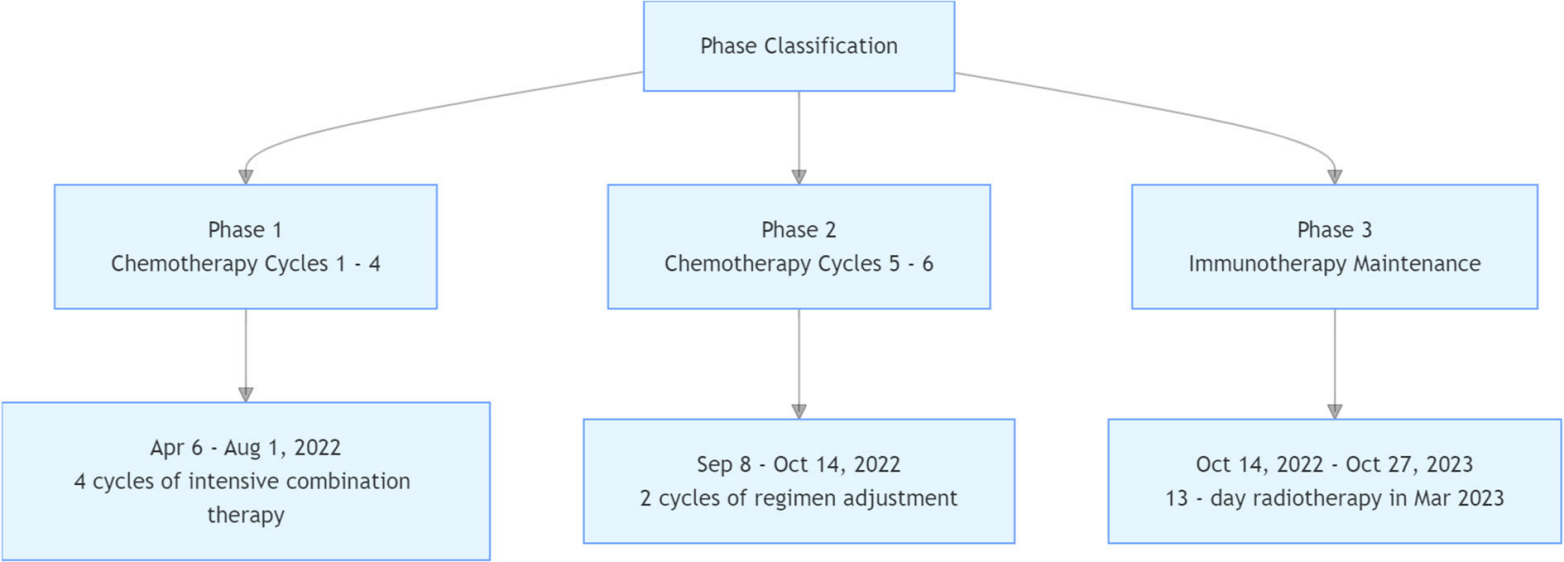
Treatment protocol.

Treatment Protocol (**Figure 4**)

Cycles 1–4 (May 6, June 16, July 12, August 1, 2022): Albumin-bound paclitaxel (200 mg on days 1 and 5), capecitabine (1.5 g twice daily on days 1–14), and subcutaneous envafolimab (400 mg on day 1).
Cycles 5–6 (September 8, October 14, 2022): Capecitabine (1.5 g twice daily on days 1–14) plus subcutaneous envafolimab (400 mg on day 1).
Maintenance phase (October 14, 2022–October 27, 2023): Subcutaneous envafolimab (400 mg every 21 days).

A follow-up PET-CT scan (February 23, 2023) revealed residual hypermetabolic activity in the left lung tumor (**Supplementary Materials**), prompting initiation of radiotherapy (gross tumor volume [GTV]; planning target volume [PTV]: GTV + 8 mm margin; total dose: 56 Gy(8 Gy per fraction, for a total of 7 fractions), March 13–25, 2023). Subsequent imaging showed complete remission of all other lesions.

Immunotherapy maintenance was discontinued after 12 months, following confirmation of a complete metabolic response on serial FDG-PET/CT (per RECIST v1.1) and sustained disease control for >18 months. This decision balanced the durable clinical benefit against the cumulative risk of immune-related toxicities from prolonged therapy, including documented treatment-associated cardiotoxicity (left ventricular ejection fraction [LVEF] 42% and B-type Natriuretic Peptide 1409.889pg/ml). Although the patient had no clinical manifestations or symptoms, we still consulted the Department of Cardiovascular Medicine. Following the advice of the Department of Cardiovascular Medicine, we administered oral medications such as bisoprolol, spironolactone, and empagliflozin. In subsequent re-examinations, the patient’s LVEF fluctuated between 38% and 50%, the BNP slowly decreased to 630.429 pg/ml, and the patient still had no clinical manifestations or symptoms.

Surveillance imaging in January 2024 showed no evidence of recurrence or metastasis. However, a PET-CT scan on February 8, 2025, demonstrated recurrent ovarian cancer, prompting reinitiation of combined immunotherapy and chemotherapy. At the time of manuscript submission, the patient had achieved an overall survival of 33 months with preserved functional status.

## Discussion

Advances in cancer screening, diagnostic imaging, and therapeutic technologies have significantly improved the survival of cancer patients, leading to a gradual increase in the incidence of multiple primary malignant neoplasms (MPMNs), which is reported to range from 0.4% to 11.7% in recent literature (1, 2). MPMNs are classified into synchronous (diagnosed within 6 months) and metachronous (diagnosed beyond 6 months) subtypes based on the interval between the onset of different primary tumors (3). Synchronous MPMNs themselves are rare, and cases involving four concurrent primary malignancies are extremely scarce—prior to this report, only one such case had been documented in the global literature (4). This case of a 71-year-old female with four synchronous primary tumors (colorectal adenocarcinoma, ovarian carcinoma, lung adenocarcinoma, and gastric cardia adenocarcinoma) who achieved a 33-month overall survival (OS) following immunotherapy-based comprehensive treatment not only enriches the clinical data on ultra-rare MPMNs but also provides valuable insights into the application of immunotherapy in this population.

The occurrence of MPMNs is closely associated with multiple factors, including genetic predisposition, immune dysfunction, environmental exposures, and prior oncologic treatments (5, 6). In this case, the patient had no family history of genetic disorders, and germline genetic testing ruled out pathogenic variants in Lynch syndrome-related genes (MLH1, MSH2, MSH6, PMS2) and other cancer susceptibility genes (APC, BRCA1/2, TP53), indicating a sporadic etiology. Notably, the patient’s long-term vegetarian diet and potential malnutrition(Hemoglobin: 59.00 g/L; Prealbumin: 15.3 mg/dL; Albumin: 34.7 g/L) may have contributed to immune compromise, which is consistent with the growing evidence linking immune dysregulation to the development of MPMNs (6). Previous studies have shown that chronic immune deficiency can impair the body’s ability to eliminate malignant cells, increasing the risk of multifocal carcinogenesis (7), which may explain the synchronous occurrence of four primary tumors in this patient.

From an epidemiological perspective, synchronous MPMNs account for approximately 10%-30% of all MPMNs (8), and the coexistence of four primary tumors is an extremely rare event. Testori et al. (3) reported the first case of four synchronous primary malignancies (breast, colon, kidney, and skin) in 2015, but the patient did not receive immunotherapy and had a limited survival time. In contrast, the 33-month OS achieved in our case highlights the transformative potential of immunotherapy for this ultra-rare subgroup. Additionally, the patient’s tumors were distributed across multiple systems (digestive, respiratory, reproductive), which differs from the more common organ-specific clustering of MPMNs (e.g., head and neck, gastrointestinal tract) (9). This systemic distribution further supports the role of systemic factors (such as immune dysfunction) in the pathogenesis of MPMNs, rather than local “field cancerization” (a mechanism often implicated in organ-specific MPMNs) (10).

The management of synchronous MPMNs requires individualized strategies based on tumor resectability, patient performance status, and molecular characteristics (7, 8). For resectable tumors, radical resection remains the cornerstone of treatment; however, for patients with multiple unresectable tumors involving multiple organs (as in this case), radical surgery is often not feasible due to excessive morbidity and mortality risks. Traditional chemotherapy and radiotherapy have limited efficacy in MPMNs due to tumor heterogeneity and cumulative toxicity (11), making novel therapeutic modalities such as immunotherapy highly anticipated.

The selection of immunotherapy for this patient was guided by key molecular characteristics: next-generation sequencing (NGS) revealed microsatellite instability-high (MSI-H) status in the colorectal tumor, with a tumor mutational burden (TMB) of 8 mutations/Mb. MSI-H/dMMR is a well-established predictive biomarker for immune checkpoint inhibitor (ICI) response, as defective mismatch repair leads to the accumulation of neoantigens, enhancing tumor immunogenicity (12). Envafolimab, the world’s first subcutaneously administered PD-L1-targeting single-domain antibody (sdAb), was approved in China in 2021 for the treatment of unresectable or metastatic MSI-H/dMMR solid tumors (13). Its unique structure—combining a PD-L1-specific sdAb with the Fc fragment of human IgG1—confers high stability, solubility, and an extended serum half-life, addressing the short half-life limitation of conventional sdAbs (14). Clinical data from a single-arm meta-analysis showed that envafolimab achieved a favorable objective response rate (ORR) and disease control rate (DCR) in MSI-H/dMMR solid tumors, with manageable safety profiles (13), supporting its use in this patient.

The combination of immunotherapy with chemotherapy (albumin-bound paclitaxel + capecitabine) in the induction phase was also a rational choice. Chemotherapy can enhance antitumor immunity by inducing immunogenic cell death, releasing neoantigens, and reducing immunosuppressive cells in the tumor microenvironment (15). For MPMNs with high heterogeneity, chemotherapy may help control rapidly progressing lesions while immunotherapy exerts a sustained antitumor effect. In this case, the combination regimen achieved effective disease control in all four tumors, with most lesions reaching complete remission after maintenance therapy—consistent with previous reports that ICI-chemotherapy combinations improve outcomes in advanced solid tumors (16). The addition of radiotherapy for residual lung lesions further highlights the value of multimodal therapy: radiotherapy not only directly ablates local residual tumor but also induces abscopal effects, potentially enhancing systemic antitumor immunity (17).

The 33-month OS achieved by this patient is clinically significant, especially considering the poor prognosis of synchronous MPMNs reported in previous studies. A population-based study of MPMNs found that the median OS of patients with synchronous tumors was only 15–20 months (18), while this patient’s survival nearly doubled that figure, underscoring the potential of immunotherapy to improve outcomes in this high-risk population. Notably, the patient maintained a good functional status throughout treatment, with only grade 2 cardiotoxicity (LVEF 42%)—a manageable adverse event that resolved with supportive care. This aligns with the safety profile of envafolimab observed in clinical trials, where immune-related adverse events (irAEs) were mostly mild to moderate (13). However, it also reminds clinicians of the need for close monitoring of long-term irAEs during immunotherapy maintenance, especially in elderly patients with multiple comorbidities.

The recurrence of ovarian cancer after 27 months of initial treatment warrants further discussion. Although the patient achieved durable disease control with immunotherapy, ovarian cancer is inherently aggressive, and resistance to ICIs may develop over time. Possible mechanisms of resistance include loss of MSI-H status, downregulation of PD-L1 expression, or alterations in the tumor immune microenvironment (19). The decision to reinitiate combined immunotherapy and chemotherapy for recurrent disease is supported by evidence that retreatment with ICIs can yield clinical benefits in previously responsive patients (20).

Despite the encouraging outcomes of this case, significant challenges remain in the management of MPMNs. First, there is a lack of standardized treatment guidelines due to the rarity of the disease and the absence of randomized controlled trials. Most current evidence is derived from case reports and small retrospective studies (1, 4), highlighting the need for multicenter collaborative research to accumulate more data. Second, the identification of predictive biomarkers for immunotherapy in MPMNs requires further exploration. While MSI-H/dMMR is a validated biomarker, most MPMNs are microsatellite stable (MSS) (18), and novel biomarkers (e.g., TMB, PD-L1 expression, gut microbiota) need to be evaluated to guide treatment decisions in this larger subgroup. Third, the optimal combination and sequencing of therapies (immunotherapy, chemotherapy, radiotherapy, targeted therapy) remain unclear. For example, whether targeted therapy can be integrated into the regimen for MPMNs with actionable mutations (e.g., EGFR mutations in lung cancer (21), BRCA mutations in ovarian cancer (22)) requires further investigation.

In addition, the diagnosis of synchronous MPMNs remains a clinical challenge, as these tumors are often misdiagnosed as metastatic disease due to their temporal and spatial clustering (23, 24). This case emphasizes the importance of comprehensive evaluation using multiple modalities (PET-CT, endoscopy, pathological biopsy, NGS) to distinguish primary tumors from metastases. For example, immunohistochemical profiling (TTF-1+/NapsinA+ in lung cancer, CK20-/CDX-2- in ovarian cancer) and site-specific pathological features were critical for confirming the independence of the four tumors. This case provides a novel direction for immunotherapy combination in inoperable synchronous MPMNs, while also highlighting research gaps in this field regarding pathogenesis, predictive biomarkers of efficacy, standardized treatment regimens, and other aspects. Subsequent multi-center studies, multi-omics analyses, and clinical trials are required to further validate and optimize relevant diagnosis and treatment strategies.

## Conclusion

This case report describes the first documented long-term survival of a patient with four synchronous primary malignant neoplasms treated with envafolimab-based immunotherapy, providing valuable insights into the management of this ultra-rare disease. The findings suggest that immunotherapy, particularly in MSI-H/dMMR MPMNs, can achieve durable disease control and improve survival when combined with chemotherapy and radiotherapy. Multidisciplinary team (MDT) collaboration, thorough molecular profiling, and individualized multimodal therapy are key to optimizing outcomes. Future research should focus on multicenter studies to validate the efficacy of immunotherapy in MPMNs, identify novel predictive biomarkers, and develop standardized treatment algorithms—ultimately improving the prognosis of this challenging patient population.

## Data Availability

The original contributions presented in the study are included in the article/**Supplementary Material**. Further inquiries can be directed to the corresponding author.
