# Supplementary material for: Case Report: Multimodal immunotherapeutic regimen (envafolimab + chemotherapy + radiotherapy) for four synchronous primary malignant neoplasms: a case report of 33-month survival and implications for tumor immunology
Source: Front Oncol. 2026 Jan 8;15:1727323. doi: 10.3389/fonc.2025.1727323 (PMC12823495; doi:10.3389/fonc.2025.1727323)
Supplement: Supplementary file 1 [file DataSheet1.docx]

Supplementary files


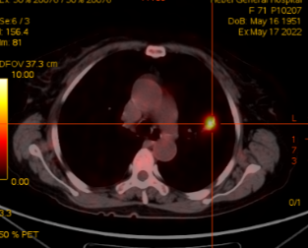

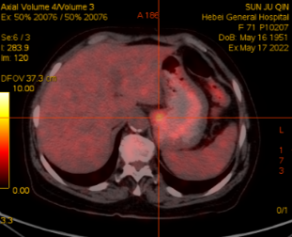

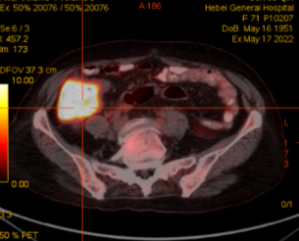

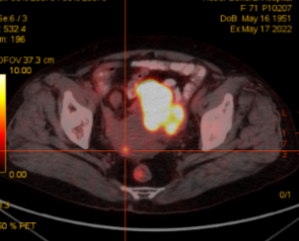


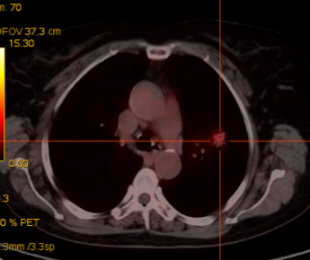

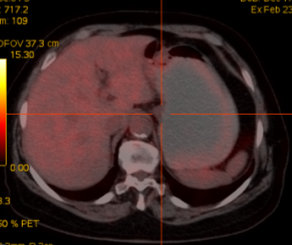

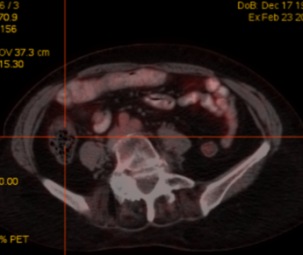

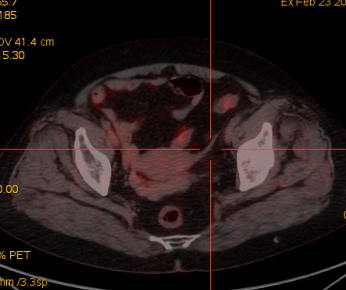


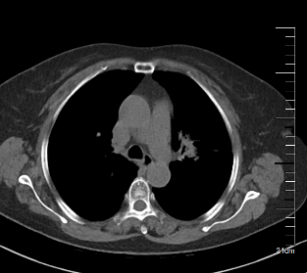

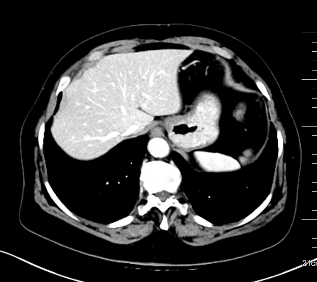

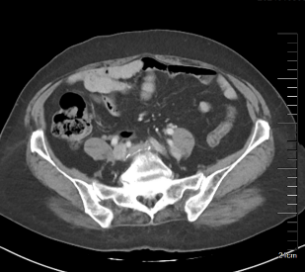

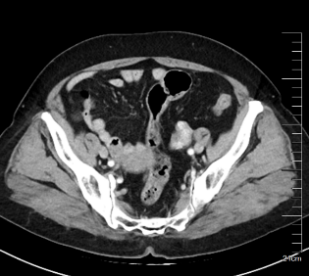


First line:

PET-CT prior to treatment (May 23, 2022): A hypermetabolic nodule in the apical-posterior segment of the left upper lung lobe, measuring approximately 22mm×21mm, with lobulation, elongated spiculation, pleural retraction, and bronchial truncation. Focal hypermetabolic thickening of the gastric wall in the cardia region, measuring approximately 17mm×12mm. Hypermetabolic wall thickening involving the entire layer of the ileocecal region, measuring approximately 60mm×52mm×72mm, with adjacent fat stranding and a hypermetabolic enlarged lymph node medially. An irregular hypermetabolic mass in the left pelvic cavity, measuring 72mm×60mm×63mm, and multiple nodular hypermetabolic foci on the surface of pelvic bowel loops.

Second line:

Follow-up PET-CT (February 24, 2023): The previously observed hypermetabolic ileocecal wall thickening, adjacent hypermetabolic lymph nodes, left pelvic hypermetabolic soft tissue mass, multiple nodular hypermetabolic foci in the pelvic bowel loops/peritoneum, and focal gastric cardia hypermetabolic thickening (from the prior PET-CT on May 17, 2022) have resolved, showing normalized morphology, density, and metabolism. The hypermetabolic nodule in the apical-posterior segment of the left upper lung lobe remains stable in size (24mm×20mm), with reduced metabolic activity and persistent imaging features (lobulation, elongated spiculation, pleural retraction, and bronchial truncation). Overall findings suggest partial metabolic response (PMR) post-treatment.

Third line:

Follow-up CT (January 4, 2024): The left upper lung lobe carcinoma lesion measures approximately 30mm×19mm, reduced compared to the prior study (August 23, 2023). Other lesions show no significant interval changes. Stable disease is considered.
